# Antecedents to Caregiving Differences in Black and White Families

**DOI:** 10.1093/geroni/igaa057.3336

**Published:** 2020-12-16

**Authors:** Kristie Wood, Yee To Ng, Meng Huo, Karen Fingerman

**Affiliations:** 1 University of Texas, Austin, Austin, Texas, United States; 2 University of California, Davis, Davis, California, United States

## Abstract

Researchers have observed racial differences in midlife adults’ caregiving for aging parents. Black adults typically provide more parental caregiving and report greater rewards in doing so. We asked whether Black and White young adults differ in their support to midlife parents, and furthermore, whether this support differs based on parental gender. We also examined cultural beliefs and rewards of providing support underlying racial differences in support to parents. Black and White young adults from the Family Exchanges Study II (2013; aged 18–30 years; n=114 Black and, n=358 White) reported support provided to parents, and beliefs and rewards associated with support. We assessed 6 types of support (emotional, advice, listening to talk, socializing, practical, and financial) to each parent rated 1 = once a year or less often to 8 = everyday. Multilinear models revealed that Black young adults gave significantly more support to parents than White offspring, and these racial differences were mediated by filial obligation beliefs. Compared to White young adults, Black offspring provided more frequent support to their mothers and reported that it was more rewarding, and endorsed more negative relationship quality with mothers than with fathers. Research has shown that involvement correlates with conflict, which may underlie these findings. Further, compared to White offspring, Black young adults show significantly more behaviors that lead to caregiving in later life, and there are nuanced gender differences within Black parent-child relationships, which may need to be better understood to support Black caregivers.

